# Iron Oxide Films Prepared by Rapid Thermal Processing for Solar Energy Conversion

**DOI:** 10.1038/srep40500

**Published:** 2017-01-16

**Authors:** B. Wickman, A. Bastos Fanta, A. Burrows, A. Hellman, J. B. Wagner, B. Iandolo

**Affiliations:** 1 Department of Physics, Chalmers University of Technology, SE-42196 Göteborg, Sweden; 2 Center for electron nanoscopy, Technical University of Denmark, DK-2800 Kgs. Lyngby, Denmark

## Abstract

Hematite is a promising and extensively investigated material for various photoelectrochemical (PEC) processes for energy conversion and storage, in particular for oxidation reactions. Thermal treatments during synthesis of hematite are found to affect the performance of hematite electrodes considerably. Herein, we present hematite thin films fabricated *via* one-step oxidation of Fe by rapid thermal processing (RTP). In particular, we investigate the effect of oxidation temperature on the PEC properties of hematite. Films prepared at 750 °C show the highest activity towards water oxidation. These films show the largest average grain size and the highest charge carrier density, as determined from electron microscopy and impedance spectroscopy analysis. We believe that the fast processing enabled by RTP makes this technique a preferred method for investigation of novel materials and architectures, potentially also on nanostructured electrodes, where retaining high surface area is crucial to maximize performance.

Solar-to-chemical energy conversion is expected to play an ever-increasing role in satisfying our energy needs, as most scenarios contemplate reducing and eventually abandoning the use of fossil fuels^1^,^2^. Energy from the Sun can be stored in chemical bonds, i.e. by synthesizing fuels (for instance hydrogen and methanol), in a few different schemes^3^,^4^,^5^. Direct energy conversion and storage using photocatalytic or photoelectrochemical (PEC) devices is one attractive route in this respect. The n-type semiconductor hematite (α-Fe_2_O_3_, α omitted hereinafter for simplicity) is a promising material to carry out half-cell oxidation reactions^6^, in particular water oxidation^7^,^8^,^9^ (also called oxygen evolution reaction, OER). The complimentary reduction reaction can lead to synthesis of hydrogen, but also of other molecules such as ammonia or methanol. The most advantageous properties of Fe_2_O_3_ are: (i) a bandgap energy that allows for absorption of up to 40% of the solar spectrum^10^; (ii) favourable position of the top of the valence band with respect to the thermodynamic electrochemical potential of water oxidation^11^; (iii) high availability; and (iv) high resistance to (photo)corrosion in neutral and alkaline electrolytes^12^. Among the efficiency limiting factors for this material we find: (i) an order of magnitude mismatch between photon absorption length and collection length for minority carriers (holes)^13^; (ii) a small photovoltage compared to the value of optical bandgap resulting in weaker-than-ideal oxidative power of holes^14^; and (iii) rather large overpotentials needed to initiate water oxidation^15^.

Fe_2_O_3_ is synthesized in the laboratory using a wide range of chemical and physical processes. The microstructure and composition resulting from the chosen synthesis route have a crucial impact on the PEC properties^7^,^16^. A so far unavoidable fabrication step consists of thermal oxidation/calcination in order to convert Fe or Fe hydroxides into Fe_2_O_3_^9^. Moreover, a second annealing step is performed at temperatures up to 750 °C or 800 °C, in order to maximize performance^8^. These heating steps are usually carried out in a tube or box furnace, a procedure that has the following disadvantages: (i) large temperature gradients, thus making temperature accuracy and control problematic; (ii) contamination from other materials, unless high purity dedicated furnaces are used; and (iii) relatively long ramp-up times. An alternative annealing technique is rapid thermal processing (RTP), where these issues are much less prominent^17^. In RTP, an array of lamps is used to generate heat^18^, and the emitting power of the lamps delivers high temperature accuracy at the specimen. Ramp-up rates are normally of the order of 10 °C s^−1^, but can also be as high as 400 °C s^−1^. This results in considerable time (and energy) savings as compared with tube/box furnace annealing. RTP is most often employed on semiconductors^19^ – in particular on Si and the III-V groups^20^,^21^,^22^,^23^, in order to generate oxide layers or induce doping, or on functional oxides such as YBCO^24^.

In this work, we report our first characterization of Fe_2_O_3_ films fabricated by a *one-step* RTP of Fe films as a model system for oxidation half-reactions, with a focus on O_2_ evolution from water splitting. Fe_2_O_3_ thin films are known to show a lower performance as compared to nanostructured photoanodes (such as “cauliflower-like” electrodes^25^) characterized by a larger surface area. Nonetheless, thin films are a more suitable system to study (photon induced) electrochemical processes since it is less difficult to interpret the experimental results^26^. We have focused on characterizing the physical and electrochemical properties of Fe_2_O_3_ films oxidized at different temperatures. First results indicate that one-step oxidation at 750 °C yields films with the best performance towards water oxidation. Furthermore, our physical and electrochemical characterization of the electrodes indicates a strong correlation between properties such as grain size and majority charge carrier density and PEC performance.

## Results and Discussion

### Physical characterization

**Figure 1:**
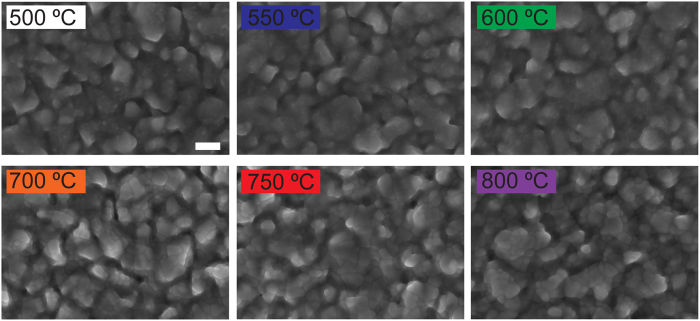
Scanning electron microscopy. SEM images of Fe_2_O_3_ films fabricated on FTO-coated glass at different oxidation temperature, *T*_*ox*_. Scale bar is 200 nm. All films are dense and polycrystalline.

We deposited Fe films by physical vapour deposition (PVD) on top of fluorine doped tin oxide (FTO) covered glass – see Methods section for fabrication details. Then, we employed RTP to oxidize the Fe into Fe_2_O_3_. In previous work^27^,^28^, electrodes with a Fe_2_O_3_ thickness of 40 nm converted from Fe at 350 °C in a flow furnace yielded highest performance. In this work, we retained the Fe_2_O_3_ thickness of 40 nm and investigated the effect of varying the oxidation temperature *T*_*ox*_ in an RTP step on the physical and PEC properties of the fabricated electrodes. Optimization of the Fe_2_O_3_ thickness and the oxidation time *t*_*ox*_ will be the objective of a future investigation. Figure 1 shows scanning electron microscope (SEM) images of films prepared using *T*_*ox*_ in a range from 500 °C to 800 °C. All the films are dense and polycrystalline, in agreement with previous reports on Fe_2_O_3_ fabricated by furnace oxidation of Fe films^29^.

**Figure 2:**
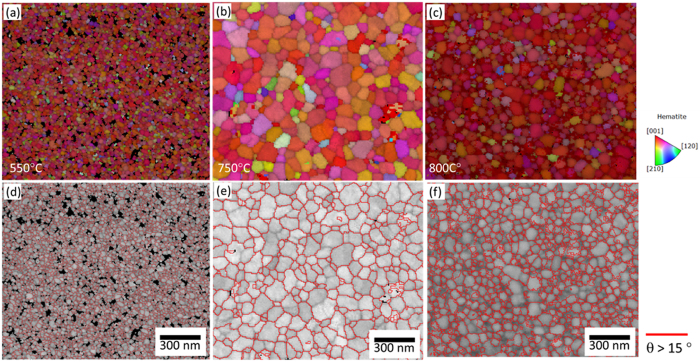
TKD characterization. Inverse pole figure maps (**a** to **c**) and pattern quality maps overlap with grain boundary map (**d**–**f**) from three hematite films oxidized at 550 °C (**a** and **d**), 750 °C (**b** and **e**) and 800 °C (**c** and **f**). Red boundaries in (**d**–**e**) reveals misorientation higher than 15°.

To characterize the microstructure of the films in greater detail, we used Transmission Kikuchi Diffraction (TKD). TKD is a novel alternative to electron backscatter diffraction (EBSD) for electron transparent samples that allows an order of magnitude improvement in spatial resolution^30^,^31^, while using a near identical setup as for EBSD. The specimen is placed at a working distance varying between 3 and 5 mm and tilted (typically between 20° and 40°) from the EBSD detector. Kikuchi patterns are captured and indexed automatically by dedicated software allowing a very high throughput. Figure 2(a–c) shows inverse pole figure maps acquired from films with *T*_*ox*_ of 550 °C, 750 °C and 800 °C, respectively. Hematite is the only phase that could be indexed for all samples. It is clear that the majority of the grains on all three films grow with a preferential orientation, [001]//growth direction, which is favourable for electronic conductivity in Fe_2_O_3_^32^. Furthermore, the 750 °C and 550 °C samples display the largest and lowest average grain size, respectively. Figure 2(d–f) shows the grayscale pattern quality map (darker gray here means worse pattern quality), overlapped with a high angle grain boundary map (where high angle grain boundaries are boundaries with a misorientation greater than 15°). It has been shown that a larger grain size and lower density of high angle grain boundaries are beneficial to the PEC properties of Fe_2_O_3_^25^.

**Figure 3:**
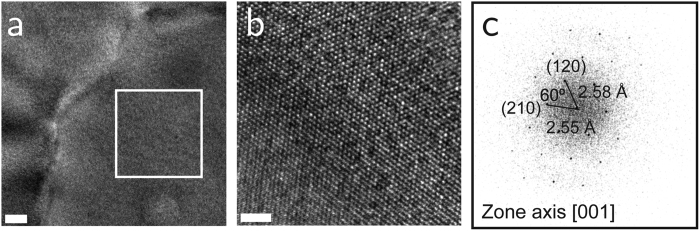
Transmission electron microscopy imaging. (**a**) Low-magnification bright field TEM image of a 40 nm Fe_2_O_3_ film (*T*_*ox*_ = 750 °C). Scale bar: 100 nm. (**b**) HRTEM image of the region enclosed by the white square in panel (a). Scale bar: 2 nm. (**c**) fast Fourier transform of the image in the panel. The diffractogram is characteristic of an orthorhombic crystal with [001] as the zone axis. The reflections corresponding to the families of planes {210} and {120} are indicated.

Figure 3a shows a low magnification bright field transmission electron microscope (TEM) image of a 750 °C film. Lattice fringes are visible in the high resolution TEM (HRTEM) image in Fig. 3b. The fast Fourier transform (FFT) of this image (Fig. 3c), results in a diffractogram characteristic of an orthorhombic crystal (such as Fe_2_O_3_) with [001] as the zone axis. The reflections corresponding to the planes (210) and (120) are indicated, with their respective inter-planar distances d_210_ = 2.55 Å and d_120_ = 2.58 Å, in good agreement with the expected corresponding values for Fe_2_O_3_.

**Figure 4:**
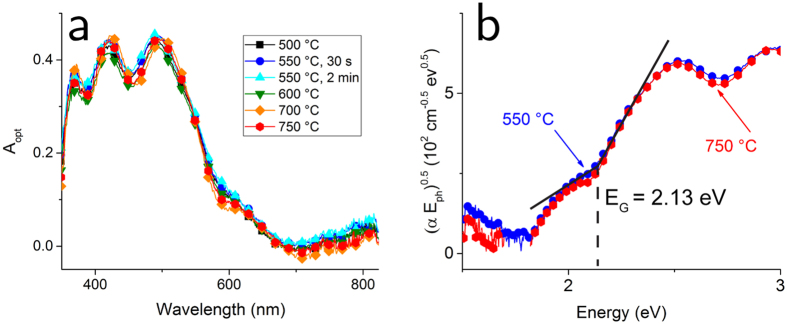
Optical characterization. (**a**) Optical absorption *A*_*opt*_ for various Fe_2_O_3_ films, measured with a spectrometer equipped with an integrating sphere. The absorption profile is almost identical for all samples. (**b**) Tauc plot for Fe_2_O_3_ films with *T*_*ox*_ = 550 °C and 750 °C. The energy of the optical bandgap *E*_*G*_ is 2.13 eV for both films.

Measurements of the optical absorption, *A*_*opt*_, reveal that it is independent of *T*_*ox*_, as shown in Fig. 4a. Starting from longer wavelengths, all samples show an absorption tail between 600 and 700 nm, which previous work has attributed to the presence of mid-gap surface states^10^. We observe a strong rise in *A*_*opt*_ starting at approximately 580 nm, and a maximum absorption of around 50% before the appearance of an interference pattern. We attribute such interference to the fact that the FTO layers have a certain non-uniformity in thickness. Having measured *A*_*opt*_ and the thickness of the films, *d*, independently, we can determine the absorption coefficient, α, and from that we can determine the optical bandgap energy, *E*_*G*_. Figure 4b shows a Tauc plot for a semiconductor characterized by an indirect bandgap with allowed transitions, i.e. (α*E*_*ph*_)^0.5^ is plotted as function of the photon energy *E*_*ph*_. Following a procedure used previously for Fe_2_O_3_ films^33^, we determine *E*_*G*_ as the energy at which the slope corresponding to the indirect bandgap absorption and the slope corresponding to the mid-gap states absorption intersect each other. We found *E*_*G*_ = 2.13 eV for both 550 °C and 750 °C samples, corresponding to a photon wavelength of 583 nm.

### PEC characterization

**Figure 5:**
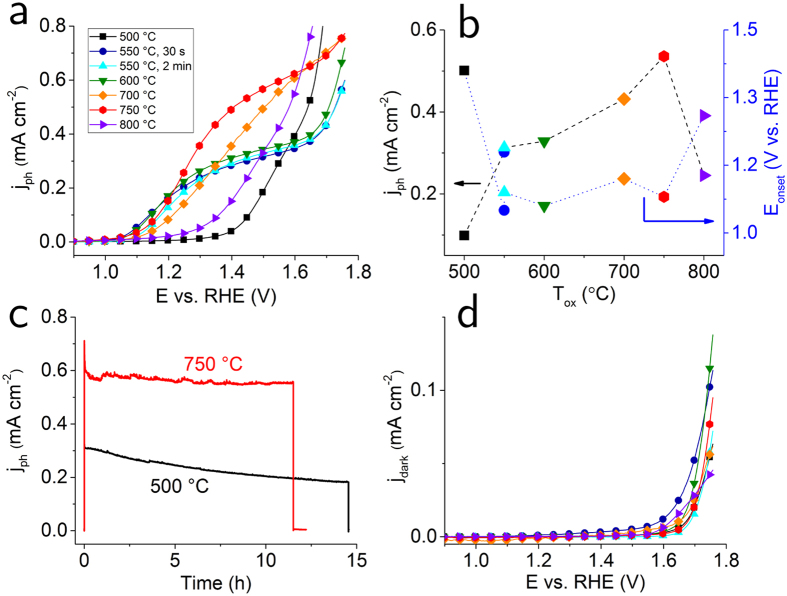
Photoelectrochemical characterization. (**a**) Photocurrent density *j*_*ph*_ as function of electrochemical potential *E* for 40 nm thick Fe_2_O_3_ films in contact with 0.1 M KOH, under 1-Sun illumination. The highest photoactivity is found for the 750 °C sample. (**b**) The values of photocurrent *j*_*ph*_ measured at *E* = 1.45 V_RHE_ (left), and of onset potential E_onset_ (right), as function of *T*_*ox*_. The symbols used are the same as for panel (a). The photocurrent reaches a maximum of 0.56 mA cm^−2^ for *T*_*ox*_ = 750 °C, then drops to 0.24 mA cm^−2^ for *T*_*ox*_ = 800 °C. Prolonging oxidation time, *t*_*ox*_, from 30 s to 2 min does not improve the performance at 550 °C. (**c**) Prolonged chronoamperometry test for 500 °C and for 750 °C samples. The 750 °C electrode shows higher stability. (**d**) Current density *j*_*dark*_ measured under the same conditions as for panel (a), except in absence of illumination.

We characterized the photocatalytic activity of the Fe_2_O_3_ films towards water oxidation by measuring the photocurrent density, *j*_*ph*_, as a function of the electrochemical potential, *E,* in a 3-electrode configuration in 0.1 M KOH electrolyte under 1-Sun illumination. The results are summarized in Fig. 5a in the form of *j*-*E* plots. Provided that the electrochemical potential scan rate is slow enough and that the material is not subject to (photo)corrosion for the duration of the measurement (in other words, that the non-Faradaic currents are much smaller than the Faradaic currents), the photocurrent is directly proportional to the amount of O_2_ evolved. We have shown that this is indeed the case for Fe_2_O_3_ films in previous work^34^.

Photoanodes prepared using *T*_*ox*_ = 500 °C show the lowest performance, both in terms of photocurrent onset potential, *E*_*onset*_ and of maximum photocurrent, *j*_*max*_, (i.e., before the onset of the current due to electrolysis without illumination). *E*_*onset*_, is around 1.4 V vs. the reversible hydrogen electrode (RHE), and *j*_*max*_ is approximately 0.4 mA cm^−2^. Increasing *T*_*ox*_ to 550 °C causes a substantial improvement in performance. *E*_*onset*_ shifts cathodically (i.e. towards more negative potentials) by about 300 mV. Neither prolonging *t*_*ox*_ from 30 s to 2 min, nor increasing *T*_*ox*_ to 600 °C has an appreciable impact on the *j*-*E* curve. For *T*_*ox*_ = 700 °C, we notice an improvement in the magnitude of *j*_*ph*_, but also a slightly less steep slope. The photoanode with *T*_*ox*_ = 750 °C shows the best performance: *E*_*onset*_ of around 1.1 V vs. RHE and *j*_*max*_ of 0.6 mA cm^−2^. Interestingly, further increasing *T*_*ox*_ to 800 °C results in a loss of photoactivity, with an anodic shift of *E*_*onset*_ to 1.3 V vs. RHE. Figure 5b shows a comparison of the photocurrent achieved at *E* = 1.45 V vs. RHE, as well as of *E*_*onset*_, as function of *T*_*ox*_. Clearly, an oxidation temperature of 750 °C results in the highest value of *j*_*ph*_, close to 0.6 mA cm^−2^. It is worth mentioning that the borosilicate glass substrates used here suffered severe cracking if kept at a certain *T*_*ox*_ for specific periods of time; longer than 2 min for *T*_*ox*_ < 700 °C, 5 s for *T*_*ox*_ < 800 °C or even for merely 2 s for *T*_*ox*_ = 800 °C. Therefore, we cannot conclude from the present set of measurements that we have reached the optimal PEC performance for a given *T*_*ox*_. This will be the object of future investigations. Nonetheless, a *j*_*ph*_ close to 0.6 mA cm^−2^ is comparable to the value measured on films of Fe_2_O_3_ with similar thickness fabricated on FTO using techniques such as sputtering^35^ or atomic layer deposition^36^ followed by furnace annealing. However, compared to these methods, RTP allows for faster and energy-saving processing. We highlight that the trends in both *j*_*ph*_ and *E*_*onset*_are consistent with the average grain size and density of grain boundaries as revealed by TKD analysis. Larger grain size in Fe_2_O_3_ is known to reduce charge recombination and thus enhance photocurrent^7^. It has also been demonstrated that high angle grain boundaries (with misorientation larger than 15°, as also defined here) can cause significant potential drops along the z-axis of the electrodes, which results in a stronger bias required to initiate water splitting, or in other words a more anodic *E*_*onset*_^25^. Figure 5c shows the results of a prolonged chronoamperometry test performed on the best and worst performing photoelectrodes. Here, we kept *E* constant while monitoring *j*_*ph*_ as function of time. While the sample oxidized at 500 °C shows a degradation of performance leading to a loss of 35% of *j*_*ph*_ after 12 h, the sample oxidized at *T*_*ox*_ = 750 °C stabilizes within one hour and remains constant over the whole measurement. Therefore, the 750 °C electrode outperforms the 500 °C electrode also in terms stability. Finally, Fig. 5d show results from *j*_*dark*_ measurements under the same conditions as for Fig. 5a, except in absence of illumination. All films show rather similar performance when acting as water oxidation electrocatalysts, with a current onset above 1.65 V vs. RHE and maximum *j*_*dark*_ of less than 0.1 mA cm^−2^.

### Impedance spectroscopy measurements

**Figure 6:**
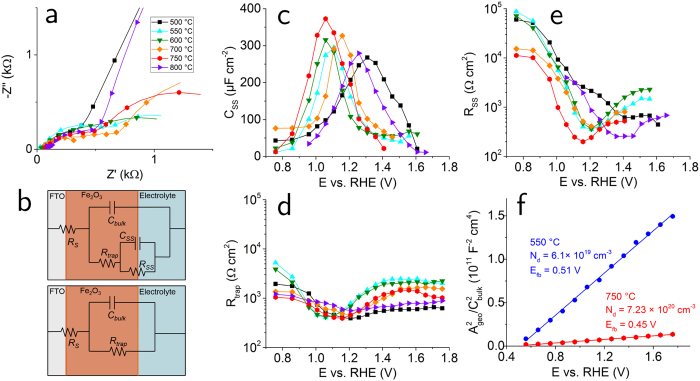
Impedance spectroscopy characterization. (**a**) Nyquist plots for Fe_2_O_3_ electrodes with different *T*_*ox*_, in contact with 0.1 M KOH, under 1-Sun illumination, at *E* = 1.16 V_RHE_. (**b**) Equivalent circuits used to fit the EIS data. Top: the Hamann equivalent circuit used to fit Nyquist plots with two time constants. Bottom: the Randle circuit used to fit plots with one time constant. Panels (c–f) show results from the data fitting. (**c**) Surface states capacitance, *C*_*SS*_. (**d**) Trapping resistance *R*_*trap*_. (**e**) Charge transfer resistance *R*_*SS*_. (**f**) Mott-Schottky analysis performed on the 550 °C and 750 °C samples.

In order to gain a deeper insight into the electrochemistry of our Fe_2_O_3_ films, we performed extensive electrochemical impedance spectroscopy (EIS). We obtained Nyquist plots (negative imaginary vs. real component of the total complex impedance *Z*) for all samples at various values of *E,* under 1-Sun illumination. Figure 6a shows Nyquist plots for different photoanodes at *E* = 1.16 V vs. RHE. The samples oxidized at 500 °C and 800 °C show a notably higher impedance than the other electrodes. This is consistent with *j*-*E* profile of Fig. 5a, since *E*_*onset*_ for these samples is more anodic than 1.16 V vs. RHE. Nyquist plots recorded between 1.76 V and 0.76 V vs. RHE for all measured samples are shown in Figure S4.

In order to perform more quantitative analysis on this data, we defined equivalent circuits (ECs) based on the physical processes occurring in the Fe_2_O_3_ and at the Fe_2_O_3_/interface during the PEC measurements. Figure 6b shows the two ECs used in this work: The Hamann equivalent circuit^36^ used to fit plots with two semi-circles, which indicate the presence of two time constants; and the Randle circuit^37^ used to fit plots with one semi-circle, characterized by one time constant. The Hamann circuit contains the following elements: the FTO-Fe_2_O_3_ contact resistance, *R*_*S*_; the resistance associated with charge recombination losses in the Fe_2_O_3_, *R*_*trap*_; the capacitance of the space-charge layer in Fe_2_O_3_ (i.e. of the region of Fe_2_O_3_ where the built-in field is present), *C*_*bulk*_; the capacitance associated with the charging and discharging of surface states, *C*_*SS*_; and the resistance associated with the transfer of charges from the surface states to the electrolyte, *R*_*SS*_. This EC was first introduced by Hamann and co-workers to describe water oxidation taking place via surface states at the Fe_2_O_3_/electrolyte interface, i.e. holes are first transferred from bulk Fe_2_O_3_ to surface states, and then to the electrolyte. The Randle circuit contains 3 elements: *R*_*S*_ in series with the parallel impedance of *C*_*bulk*_ and *R*_*trap*_. Such an EC is more appropriate for a water oxidation process where there is direct transfer of holes from the semiconductor valence band to the electrolyte, and where surface states act as spectators. Examples of Nyquist plots fitting are shown in Figure S5 for a 750 °C electrode.

Figure 6(c–e) shows the results of the analysis of EIS data. First, we turn our attention to the surface states capacitance *C*_*SS*_ in panel (c). We observed the same trend for all samples: for a given *T*_*ox*_, *C*_*SS*_ is close to zero for *E* < *E*_*onset*_; then, it quickly increases from *E* = *E*_*onset*_, reaching a maximum for *E* = *E*_*onset*_ + 150–200 mV; finally, it decreases back to negligible values for yet more anodic potentials. We observed the same behaviour in previous studies^38^,^39^, which is the fingerprint for water oxidation taking place *via* surface states. This picture is corroborated by the plot of *R*_*SS*_ in panel (b): *R*_*SS*_ is rather high (≥10^4^ Ω cm^2^) for *E* < *E*_*onset*_, indicating that water oxidation is hindered by charge recombination at the surface states. For a more anodic *E, R*_*SS*_ drops between 3 and 4 orders of magnitude and reaches a minimum for *E* = *E*_*onset*_ + 150–200 mV depending on the electrode, and it levels off for even more anodic *E*. The trapping resistance associated with charge recombination in the bulk, *R*_*trap*_, is characterized by a less pronounced variation as a function of *E*. Nevertheless, it is interesting to note that samples with relatively similar *j*-*E* plots also have similar *R*_*trap*_ profiles (550/600 °C, 700/750 °C, and 500/800 °C). We can conclude that water oxidation takes place *via* surface states for all samples independently of *T*_*ox*_. Furthermore, the data analysis indicates a strong correlation between trends in *E*_*onset*_ and C_*SS*_, as well as between *j*_*ph*_ and *R*_*SS*_.

Finally, we performed Mott-Schottky analysis to determine the flat band potential, *E*_*fb*_, and the density of charge donors *N*_*d*_ (corresponding to the charge carrier concentration in the semiconductor in the dark) for two samples. Here, we measured *Z* at a specific frequency in the dark as a function of *E*. We chose a frequency of 10^4^ Hz, in order to avoid filling and un-filling of surface states at the Fe_2_O_3_/electrolyte interface. Then, we used the Randle EC to extract values of *C*_*bulk*_ as function of *E*. The following relation for *C*_*bulk*_ can be derived from electrostatic considerations^38^:


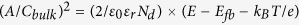


where *A* is the area of the electrode exposed to the electrolyte, *ε*_*0*_ is the permittivity of vacuum, *ε*_*r*_ is the dielectric constant of the semiconductor, *e* is the elementary electronic charge, *k*_*B*_ is the Boltzmann constant and *T* is the absolute temperature. We used here the same geometric area *A*_*geo*_we had previously used to determine *j*. We determined *N*_*d*_ and *E*_*fb*_ from the slope and from the intersection of the (*A*_*geo*_/*C*_*bulk*_)^2^ vs. *E* plot with the horizontal axis, respectively. The slope corresponding to the 750 °C electrode is much less steep than that of the 550 °C electrode, denoting a higher *N*_*d*_. In fact, the increase in *N*_*d*_ is striking, more than an order of magnitude: from 6.1 × 10^19^ to 7.23 × 10^20^ cm^−3^. While the former value is characteristic of Fe_2_O_3_ with a negligible amount of external impurities and where the major source of conduction electrons is oxygen vacancies^40^, the latter value is typical of Fe_2_O_3_ samples with external impurities acting as electron donors^41^. Several reports have described an improvement in water oxidation photocurrent on Fe_2_O_3_ upon doping with elements with higher valence than Fe, for instance Ti, Si and Sn^42^. In particular, it has been shown that for sufficiently high temperatures (typically between 725 °C and 750 °C) Sn diffuses out of FTO and into the Fe_2_O_3_, thus doping the latter and enhancing the photocurrent^43^. Indeed, XPS measurements confirmed the diffusion of Sn from FTO into Fe_2_O_3_ for *T*_*ox*_ = 750 °C, while this was not the case for *T*_*ox*_ = 550 °C (see Figure S6). We conclude that an oxidation temperature of 750 °C is sufficient to incorporate Sn in the Fe_2_O_3_ film. We therefore ascribe the enhancement of *j*_*ph*_ upon increasing *T*_*ox*_ from 550 °C to 750 °C to the combination of increased average grain size and higher majority charge carrier concentration. Finally, the 800 °C electrode shows a worse photoresponse than the 750 °C one in terms of both *E*_*onset*_ and *j*_*ph*_, despite a yet higher *T*_*ox*_, as mentioned earlier. We identified two likely mechanisms for the lower *j*_*ph*_. First, the average grain size is smaller, as evidenced by TKD analysis. Second, the FTO- Fe_2_O_3_ contact resistance *R*_*S*_ is increased, as shown in Figure S7, which results in a lower electron conductivity in the electrode. Furthermore, we can ascribe the more anodic *E*_*onset*_ to the higher density of high angle grain boundaries, which is once again clear from TKD analysis.

From the Mott-Schottky plots we also concluded that *E*_*fb*_ is slightly lower for the 750 °C sample than for the 550 °C sample (0.45 V and 0.51 V vs. RHE, respectively). The similar values of *E*_*fb*_ indicate that the amplitude of the built-in field in Fe_2_O_3_ is hardly affected by *T*_*ox*_. Further confirmation that the thermodynamics of the solid/liquid junction is to a large extent independent of *T*_*ox*_ comes from open circuit measurements both in the dark and under illumination, from which we determined that the photovoltage sustained by Fe_2_O_3_, *E*_*ph*_, is around 0.25 V for all samples (see Figure S8). While such a value is comparable with what has been recorded on other model systems based on Fe_2_O_3_ thin films^14^, it is considerably smaller than the magnitude of the optical bandgap, which highlights that there is substantial room for improvement.

### Conclusion

In summary, we presented RTP as convenient method to fabricate thin films of Fe_2_O_3_ in one-step oxidation of Fe. 40 nm thick Fe_2_O_3_ films oxidized at 750 °C delivered the maximum values of photocurrent density resulting from water oxidation. Factors contributing to such performance include: the activation of Sn dopants from the underlying FTO, as indicated by Mott-Schottky analysis; a larger average grain size and a lower density of grain boundaries, as revealed by TKD characterization. In particular, TKD has proven to be excellent for characterizing the microstructure (phase composition, crystal orientation and grain boundaries) of thin oxide films with a resolution of the order of a few nm. Further optimization of other parameters, such as Fe_2_O_3_ thickness and oxidation time, together with integration of a water oxidation catalyst are likely to lead to further enhanced performance. We consider Fe_2_O_3_ thin films an appealing candidate for top-cell photoanode material in tandem devices for photon-induced oxidation reactions in PEC devices. While we focused on Fe_2_O_3_ films in this work, RTP is readily applicable to other semiconductors of interest in the field. In particular, the fast processing enabled by RTP makes it a suitable candidate for investigation of novel materials and architectures. Finally, although we have not attempted working on nanostructured electrodes in this work, we believe that very short processing times enabled by RTP make this technique attractive for oxidation/annealing of nanostructured electrodes, which often exhibit a decrease of surface to volume ratio due to the longer thermal treatments normally used.

## Methods

### Sample fabrication

Fe_2_O_3_ thin films were fabricated on top of FTO coated borosilicate glass substrates (Techinstro, sheet resistance ≤10 Ω/square), and on TEM ‘windows’ made in-house following the procedure by Grant *et al*.^44^. First, Fe films were deposited by physical vapour deposition (PVD 225, Kurt. J. Lesker, base pressure <5 × 10^−7^ mbar), and their thickness was measured *in situ* using a quartz-crystal microbalance monitor. Then, Fe was converted into Fe_2_O_3_ by RTP in air (JIPELEC Jet First 200, halogen lamps). For each sample, the temperature was ramped up from room temperature to the respective oxidation temperature, *T*_*ox*_, in 100 s, and then kept constant for a time *t*_*ox*_ varying between 1 s and 120 s, and allowed to cool in air. The temperature was monitored during the processing using a thermocouple in contact with the back side of the substrates. Room temperature was reached again within 300 s.

### Physical Characterization

SEM imaging of the as-prepared samples was performed in a Supra 60 VP SEM (Zeiss), at an acceleration voltage of 5 kV. Films oxidized at 550, 750 and 800 °C on TEM ‘windows’ were imaged using TKD in a Nova Nano lab 600 SEM (FEI). A 20° tilt angle between the samples and the electron beam was used, together with a working distance of 3 mm. Low vacuum mode was used, with water vapour pressure of 20 to 50 Pa and a devoted low vacuum detector, in order to decrease sample contamination observed in high vacuum mode and to avoid sample drift. An accelerating voltage of 30 kV and a beam current ranging from 18 nA to 24 nA were used. TKD patterns were recorded using an e-FlashHR EBSD detector (Bruker) and analyzed using both CrystAlign (Bruker) and OIM TSL analysis software. All samples were investigated in different locations (with at least 1500 analyzed grains from each sample). In order to determine the average grain size and the density of grain boundaries, data was processed to remove uncertain points and to define a grain. A grain was defined as an area containing at least 3 data points with the same orientation and with a misorientation larger than 15° to its neighbour. All data set containing less than 3 points were removed from the maps, due to uncertainty in the indexing and are shown as black areas. High resolution bright field TEM images were acquired from the samples deposited on TEM ‘windows’, using a Titan 80–300 TEM (FEI), with a post objective lens spherical aberration corrector (CETCOR unit, CEOS) operated at an accelerating voltage of 300 kV.

The optical absorption in the photoanodes was measured using a spectroradiometer (RPS900-R, International Light), equipped with an integrating sphere (Mikropack, ISP-50-8-R-GT), coated with a polytetrafluoroethylene (PTFE) reflective coating. The absorption *A*_*opt*_ in the Fe_2_O_3_ was measured using a FTO covered substrate as reference. The absorption coefficient *α* was obtained using the relation 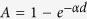, where *d* is the Fe_2_O_3_ thickness. The thickness was measured using an Easyscan v.1 atomic force microscope (Nanosurf) in tapping mode. XPS spectra were acquired in a Perkin Elmer Phi 5500 setup (base pressure <5 × 10^−10^ mbar) using Al_Kα_ radiation of 1.4866 keV. The XPS spectra were shifted using the C(1 s) peak corresponding to adventitious carbon (284.5 eV) as a reference.

### PEC measurements

All PEC characterization was carried out in a three electrode configuration in a H-type glass cell with working electrode and counter-electrode compartments separated by a glass frit. A Gamry Ref600 potentiostat was used, with a solar simulator (SKU SS150, Sciencetech. Inc.) as the illumination source. The light power density on the surface of the photoanode was adjusted to 100 mW cm^−2^ using a NIST-calibrated Si photodiode, and all the photoanodes were illuminated from the front. The photoanodes were used as the working electrode. Cu tape was used to contact the FTO with a Cu wire, and the electrodes were encapsulated using inert hot glue, which defined the active geometric area *A*_*geo*_. A Pt wire and an Ag/AgCl electrode (saturated KCl) were used as counter-electrode and reference electrode respectively. 0.1 M KOH electrolytes were prepared using high-resistance (18.2 MΩ) MilliQ water and were used within a few hours from preparation. The electrochemical potential *vs.* the Ag/AgCl reference electrode *E*_*Ag/AgCl*_ was converted into potential *vs.* RHE *E*_*RHE*_ using the Nernst equation: *E*_*RHE*_ = *E*_*Ag/AgCl*_ + *E*^*0*^_*Ag/AgCl*_ + 0.059 × pH, where *E*^*0*^_*Ag/AgCl*_ = 0.197 V at 25 °C. Cyclic voltammetry was first performed in the dark for at least 50 cycles at a scan rate of 100 mV s^−1^, in order to remove organic contaminants from the surface, and then at a scan rate of 10 mV s^−1^, both in the dark and under illumination. The measured current was divided by *A*_*geo*_ to obtain the current density *j*. The forward scans corresponding to the third cycle are plotted without resistance compensation. Steady state photocurrent density was obtained by chronoamperometry, where the voltage was changed in steps of 50 mV from more cathodic to more anodic potentials. After starting illumination, the photocurrent typically stabilized within 15 s, and the current was averaged over the last 20 s of the measurement. Open circuit voltage data was recorded for at least 20 min, both in the dark and under illumination, in order to ensure stabilization of the semiconductor/electrolyte interface. For the EIS measurements, the DC voltage was changed in steps of 50 mV from 1.76 V_RHE_ and 0.46 V_RHE_, to avoid polarization effects. An AC voltage with root mean square amplitude of 10 mV and frequency varying between 10^5^ Hz and 0.1 Hz was superimposed to the DC bias. Nyquist plots obtained under illumination were fitted using the software Echem Analyst (Gamry). Mott-Schottky analysis was performed in between 1.76 V_RHE_ and 0.46 V_RHE_, with an AC voltage with a fixed frequency of 10^4^ Hz.

## Additional Information

**How to cite this article**: Wickman, B. *et al*. Iron Oxide Films Prepared by Rapid Thermal Processing for Solar Energy Conversion. *Sci. Rep.* **7**, 40500; doi: 10.1038/srep40500 (2017).

**Publisher's note:** Springer Nature remains neutral with regard to jurisdictional claims in published maps and institutional affiliations.

## Supplementary Material

Supplementary Dataset 1
