# Supplementary material for: Iron Oxide Films Prepared by Rapid Thermal Processing for Solar Energy Conversion
Source: Sci Rep. 2017 Jan 16;7:40500. doi: 10.1038/srep40500 (PMC5238422; doi:10.1038/srep40500)
Supplement: Supplementary Dataset 1 [file srep40500-s1.pdf]

Supporting Information

Iron Oxide Films Prepared by Rapid Thermal Processing for Solar Energy Conversion

Authors: B. Wickman†, A. B. Fanta‡, A. Burrows‡, A. Hellman†, J. B. Wagner‡, B. Iandolo‡*

Affiliations:

† Department of Physics, Chalmers University of Technology, SE-42196 Göteborg, Sweden.

‡ Center for electron nanoscopy, Technical University of Denmark, DK-2800 Kgs. Lyngby, Denmark.

*Correspondence to: benian@cen.dtu.dk.

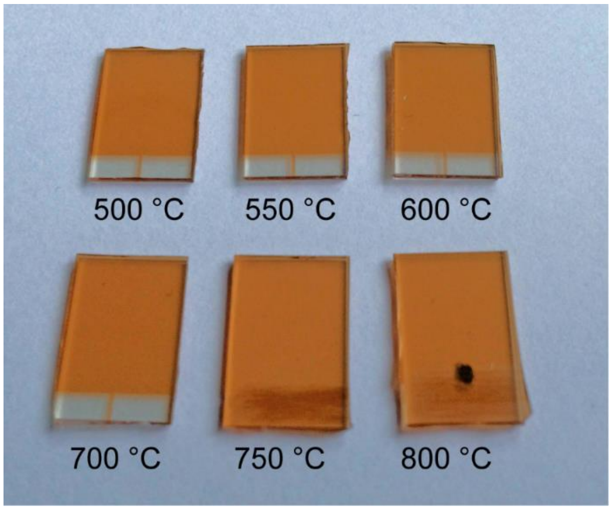

Figure S1. Photograph of Fe_2O_3 films deposited on FTO-covered borosilicate glass after oxidation by RTP at various temperatures.

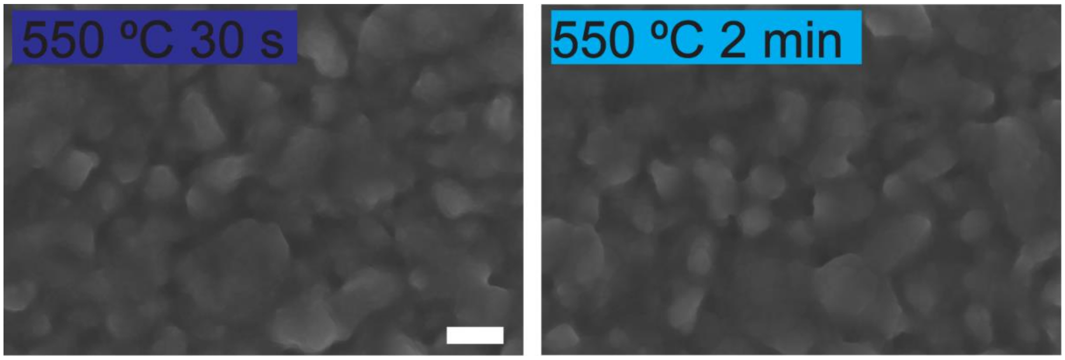

Figure S2. Comparison between top-view SEM images of Fe films oxidized at 550 °C for 30 s (left) and 2 min (right). Scale bar is 200 nm.

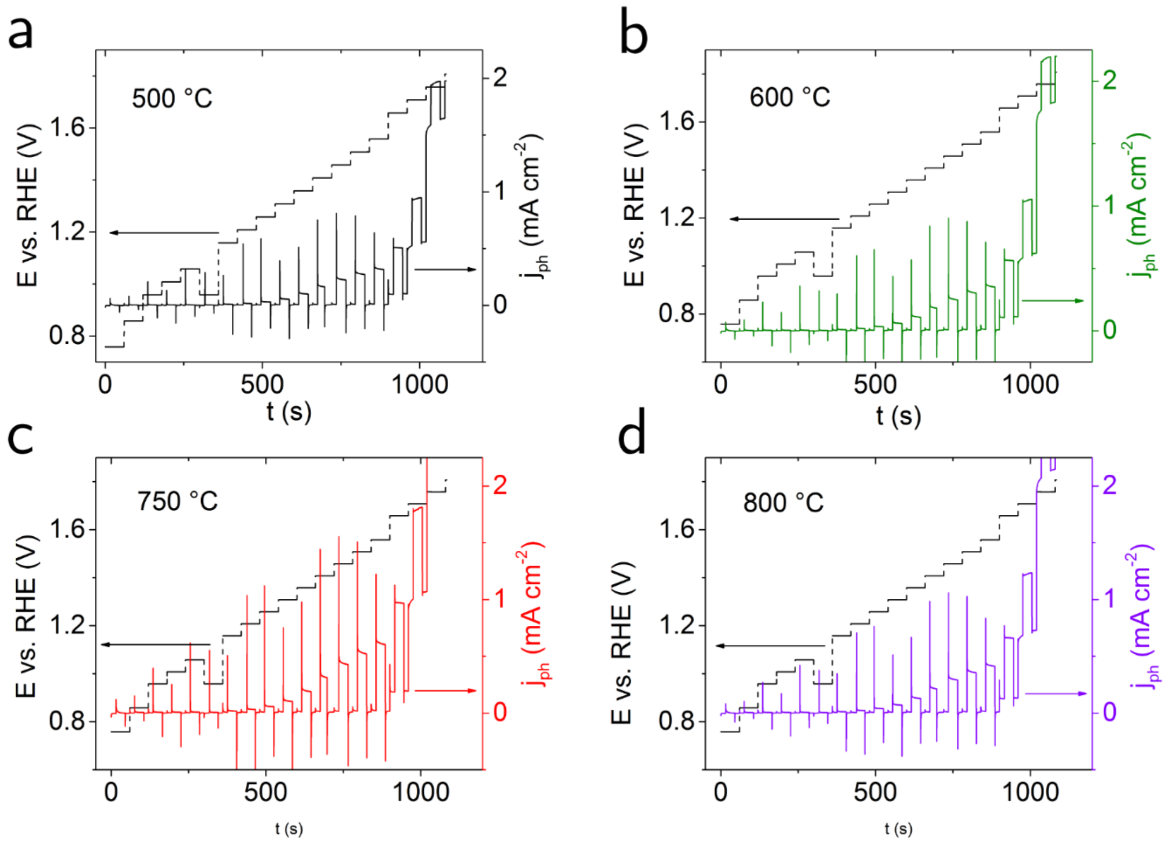

Figure S3. Chronoamperometry measurements for Fe_2O_3 in contact with 0.1 M KOH electrolyte, under 1-Sun illumination. For each panel, the broken line is the electrochemical potential E, while the continuous line is the photocurrent density j_{ph}.

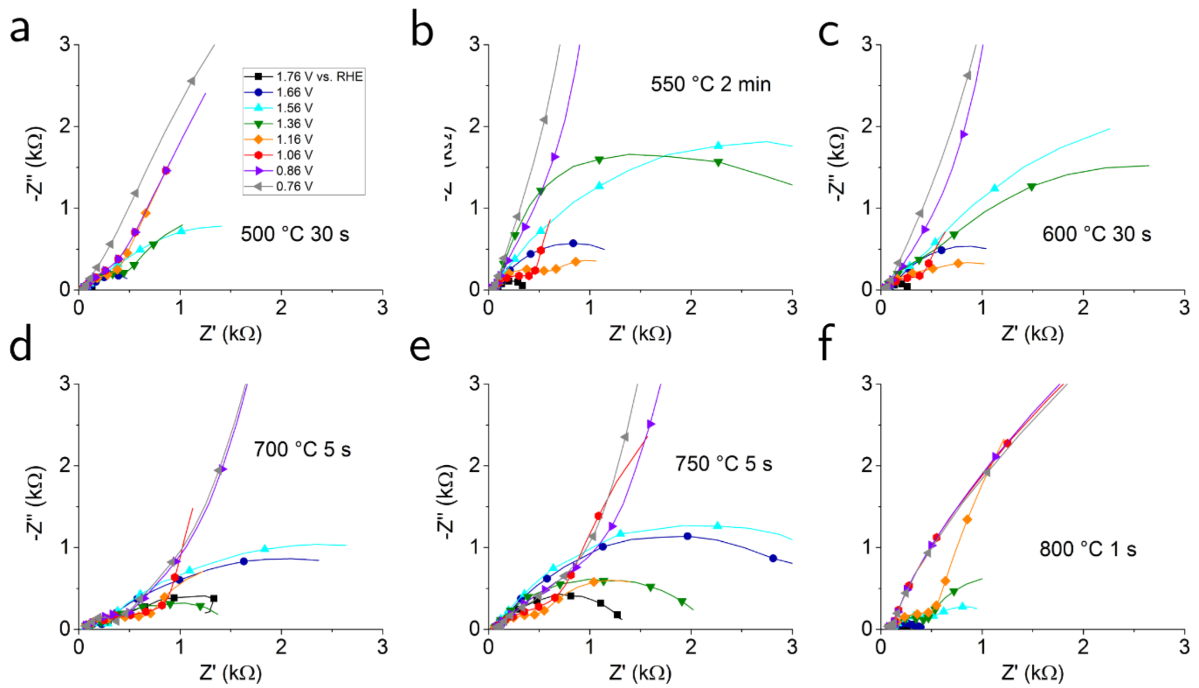

Figure S4. Nyquist plots for Fe_2O_3 films with various T_{ox} in contact with 0.1 M KOH electrolyte, under 1-Sun illumination.

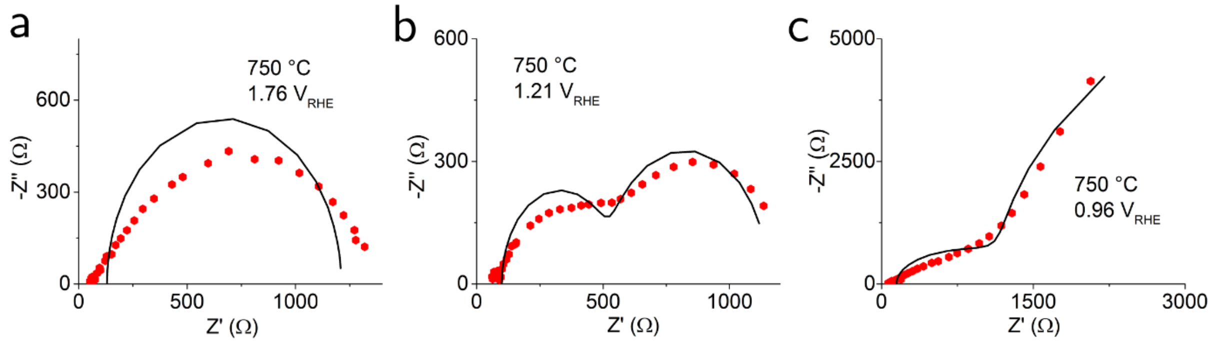

Figure S5. Fitting of Nyquist plots for a 750 °C electrode, in contact with 0.1 M KOH electrolyte, under 1-Sun illumination. Red hexagons are experimental data points, while the continuous black line is the data fit. The equivalent circuits used for the fitting (Hamann and Randle) are described in the main text.

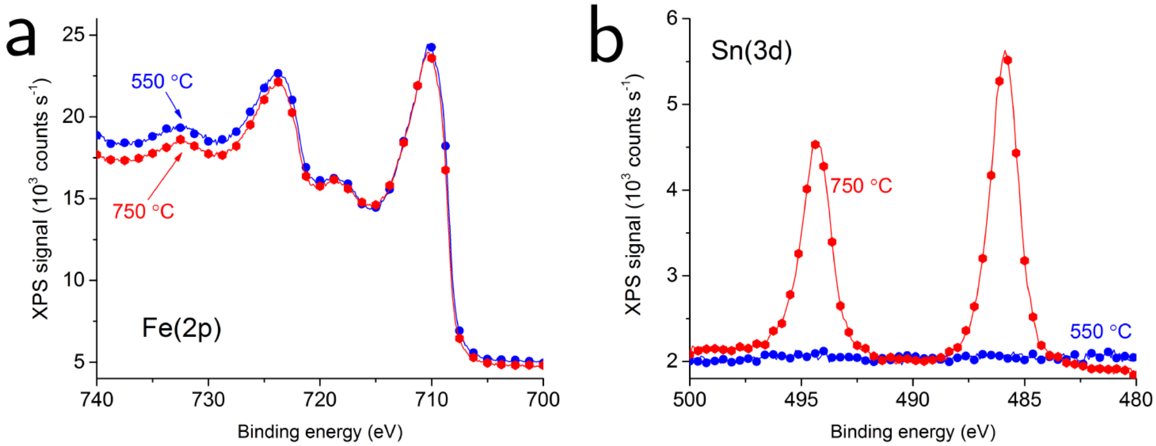

Figure S6. XPS characterization of samples oxidized at 550 °C and at 750 °C. (a) There is no clear difference between the Fe(2p) peaks of the two samples. (b) A clear Sn(3d) signature is present only in the 750 °C sample. This confirms the diffusion of Sn from FTO up to the surface of Fe_2O_3 for the 750 °C sample.

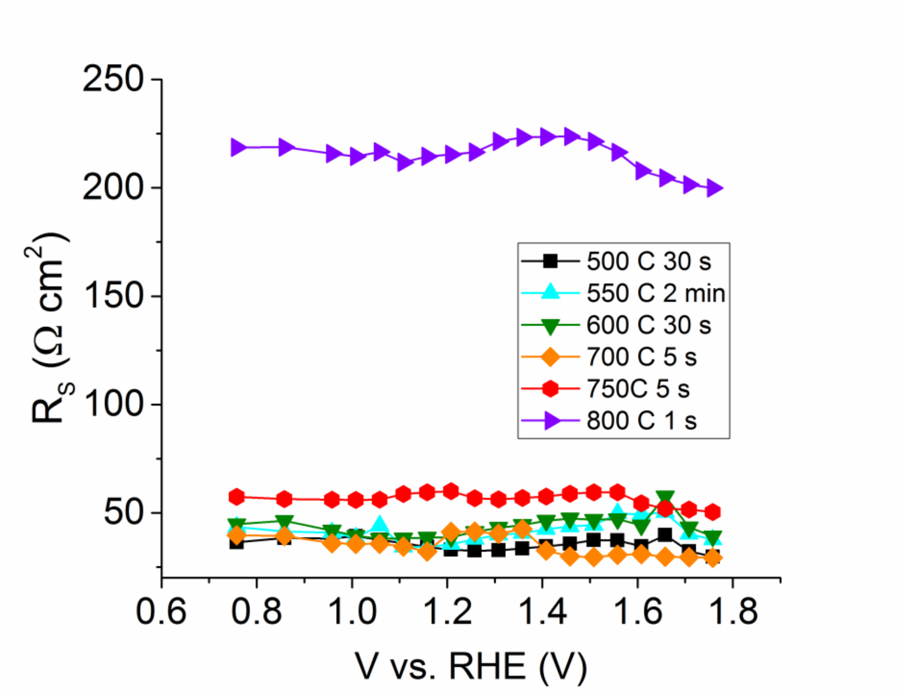

Figure S7. FTO-Fe_2O_3 contact resistance R_S for samples with varying T_{ox}, measured in contact with 0.1 M KOH electrolyte under 1-Sun illumination. The 800 °C sample clearly shows a high resistance that all the other electrodes, which indicates a loss of conductivity in the FTO electron collector layer.

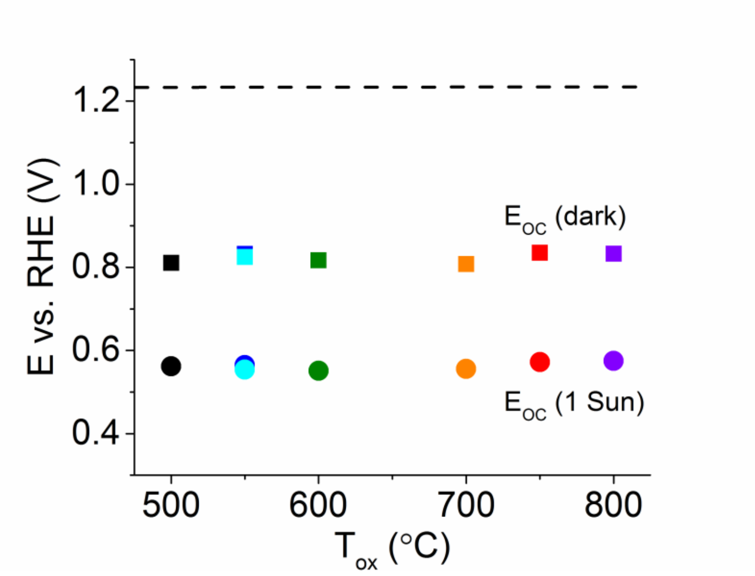

Figure S8. Open circuit voltage measurements. The difference between open circuit voltage recorded in the dark and under 1-Sun illumination determines the photovoltage E_{ph} sustained by Fe_2O_3. It is clear that E_{ph} is not affected by varying T_{ox}. The thermodynamic electrochemical potential for the oxygen evolution reaction (1.23 V against RHE) is shown by the dashed line.

Oxidation temperature T_{ox}	500 °C	550 °C	550 °C	600 °C	700 °C	750 °C	800 °C
**Oxidation time t_{ox}**	30 s	30 s	2 min	30 s	5 s	5 s	1 s

Table S1. Oxidation temperature, T_{ox}, and oxidation time for the samples investigated in this work. In particular, once T_{ox} was reached, it was kept for a time equal to t_{ox}, before cooling down to room temperature.

Oxidation temperature	550°C	750°C	800°C
**Fraction of hematite**	100%	100%	100%
**Grain size diameter (considering the number of grains)**	21 ± 10 nm	80 ± 10 nm	43 ± 10 nm
**Grain size diameter (considering the area of the grains)**	31 ± 20 nm	118 ± 10 nm	77 ± 10 nm
**Number of grains investigated**	>3000	>1500	>3000
**Fraction of grains with diameter smaller than 50 nm**	99%	38%	80%
**Fraction of grains with diameter larger than 50 nm**	1%	62%	20%
**Fraction of low angle grain boundary (LAGB)***	11%	6%	9%
**Fraction of high angle grain boundaries (HAGB)***	89%	94%	91%
**Grain boundary length/area (HAGB)**	72/µm	27/µm	54/µm
**Grain boundary length/area (LAGB)**	11/µm	2/µm	8/µm
**Fraction of indexed points (prior to data processing)**	70%	92%	72%
**Measurement step size**	5 nm	8 nm	6 nm

* LAGB means low angle grain boundaries $\theta<15°$ and HAGB means high angle grain boundaries $\theta>15°$, where θ is the misorientation angle.

Table S2: Microstructural characteristics of three hematite films investigated by TKD.
